# Quantitatively mimicking wet colloidal suspensions with dry granular media

**DOI:** 10.1038/srep10348

**Published:** 2015-06-01

**Authors:** René Messina, Sarah Aljawhari, Lydiane Bécu, Julien Schockmel, Geoffroy Lumay, Nicolas Vandewalle

**Affiliations:** 1 Laboratoire de Chimie et Physique - Approche Multi-Echelle des Milieux Complexes (LCP - A2MC) Institut de Chimie, Physique et Matériaux (ICPM), Université de Lorraine, 1 Bd. Arago, 57070 Metz, France; 2 GRASP, Physics Department, University of Liège, B-4000 Liège, Belgium

## Abstract

Athermal two-dimensional granular systems are exposed to external mechanical noise leading to Brownian-like motion. Using tunable repulsive interparticle interaction, it is shown that the same microstructure as that observed in colloidal suspensions can be quantitatively recovered at a macroscopic scale. To that end, experiments on granular and colloidal systems made up of magnetized particles as well as computer simulations are performed and compared. Excellent agreement throughout the range of the magnetic coupling parameter 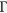 is found for the pair distribution as well as the bond-orientational correlation functions. This finding opens new ways to efficiently and very conveniently explore phase transitions, crystallization, nucleation, etc in confined geometries.

Order vs disorder in condensed matter is a fundamental aspect governing macroscopic properties^1^,^2^,^3^. In this respect, colloidal suspensions represent a model system for the physicist that enables easy visualization of microstructures in real space, establishing a direct link with the pair potential of constitutive particles. A nice illustration is provided by magnetic monolayers, thoroughly studied in the two last decades^4^,^5^,^6^,^7^,^8^. In these systems, ordering and crystallization in two dimensions were investigated experimentally and corroborated by computer simulations, when thermal agitation 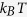 competes with particle potential energy *U*.

At first sight, granular systems made up of *macroscopic* particles possess similarities with colloidal ones. The crucial difference, however, is that thermal fluctuations are negligible for such materials. Hence granular systems are intrinsically *athermal*. Nonetheless, shaking granulates lying on vibrating substrates is a common practice to study two dimensional phase transitions^9^,^10^,^11^,^12^.

In the present study, we employ a recently proposed technique allowing a precise measurable *induced effective temperature* in two dimensional granular systems^13^. A monolayer of spherical grains is placed within a hexagonal cell which is mechanically shaken in the lateral directions, as sketched in Fig. 1(a,b). By means of Helmholtz coils, see Fig. 1(a), an external magnetic field is applied to induce repulsive interactions between neighboring particles, leading to some ordering of the grains. The mechanical agitation provides a Maxwellian distribution of the velocities of the particles as shown in Fig. 1(c). The remarkable achievement of this study^13^ was thus to establish a bridge between average particle kinetic energy 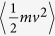 and some effective granular temperature.

The goal of this Communication is to show that the same ordering observed in delicate *wet* colloidal suspensions can be advantageously quantitatively obtained with *dry* granular systems that are much easier to manipulate. To accomplish this challenge, we will compare the experimental data of granular systems to those obtained with colloidal systems, as well as numerical Monte Carlo simulations.

In order to link colloidal and granular systems, we define a dimensionless parameter


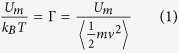


which compares the magnetic interaction *U*_m_ between particles at a typical separation and the thermal 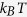 or induced kinetic energy 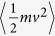. The brackets 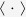 in Eq. (1) denote an ensemble average. This parameter 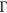 can therefore be measured in both the colloidal and granular systems (see Methods). Evidently, the magnetic coupling parameter 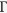 will also be a natural input parameter in our numerical simulations. It can be viewed as an inverse reduced temperature.

## Results

**Figure 1:**
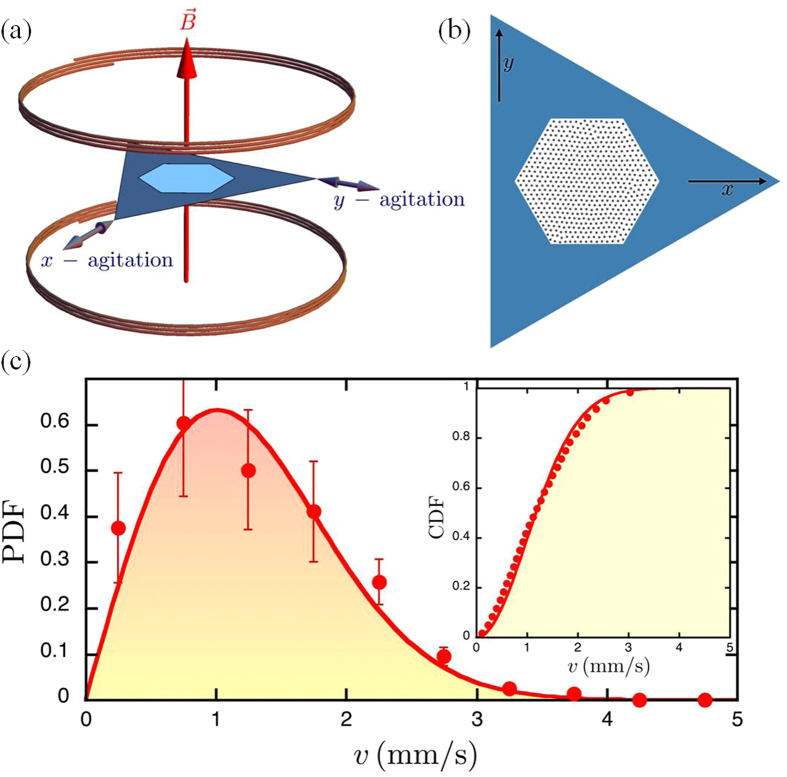
Experimental setup for the granular media and velocity distribution profile. (**a**) Sketch of the experimental setup for dry macroscopic grains. Millimetric grains of mass 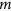 are placed in a hexagonal cell at the center of Helmholtz coils. The latter generate a perpendicular magnetic field 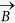 leading to repelling magnetized grains. The system is then sinusoidally excited in the 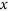 and 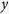 directions with the help of loudspeakers. (**b**) Top view of the cell with typical pattern. (**c**) The Probability Distribution Function (PDF) of particle velocities 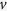 obtained at fixed magnetic field. The inset corresponds to the Cumulated Distribution Function (CDF). The solid lines represent the corresponding Maxwellian distribution^13^.

**Figure 2:**
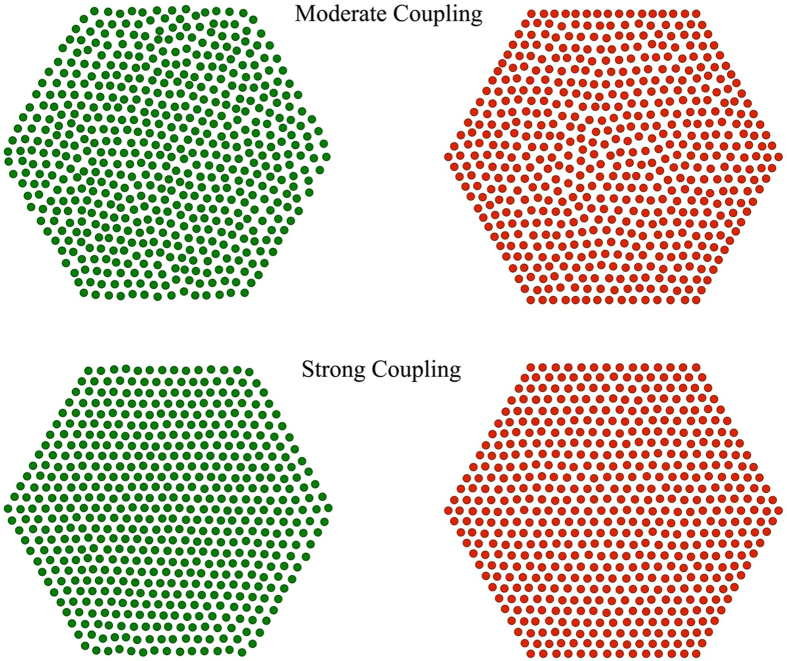
Microstructures of magnetic particles. Representative microstructures for two typical values of the magnetic coupling parameter 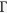: Moderate coupling (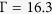) top and strong coupling (Γ = 53.5) bottom. The left panel corresponds to experimental data, whereas the right one corresponds to simulation data.

For the granular media, we use *millimetric* particles confined in a hexagonal box, as illustrated in Fig. 1. In parallel, Monte Carlo simulations are performed with the same number of particles and geometry, see Methods for more details. Comparative snapshots are displayed in Fig. 2 for different values of the magnetic coupling parameter 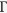. A visual inspection shows qualitatively identical ordering in experiments and simulations at given 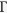. As expected the ordering increases with 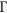 and a nearly perfect triangular lattice is recovered at the highest value of Γ = 53.5. This striking agreement is going to be analyzed now more quantitatively.

The first observable we consider to quantify the ordering is the pair distribution function 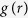 given by


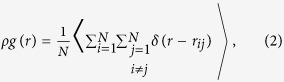


with 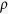 standing for the particle area density and 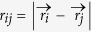 for the distance between particles 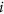 and 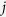 located at 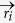 and 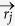, respectively. This relevant quantity 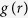 tells us about the probability of observing two particles at distance 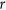.

**Figure 3:**
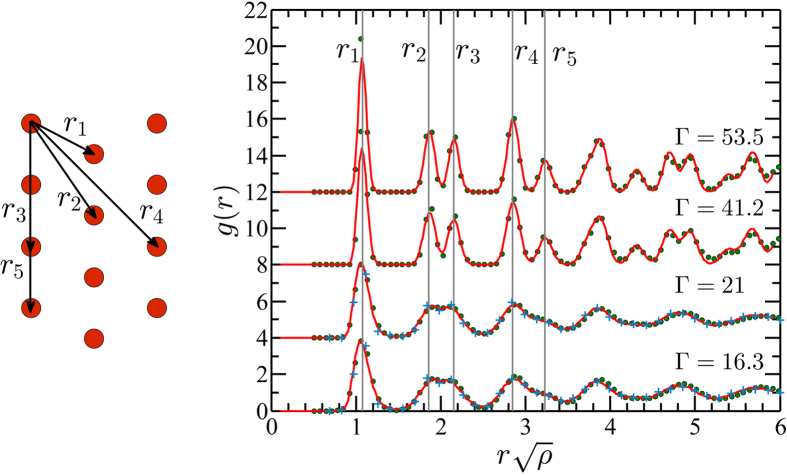
Pair distribution functions for different values of the magnetic coupling parameter 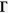
. Profiles of 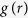 are shifted upwards upon increasing 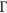 as indicated. Full lines correspond to MC simulation data, whereas dots (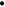) and stars (+) represent granular and colloidal experimental data, respectively. Vertical lines indicate the distances between nearest neighbors for a perfect triangular lattice as illustrated in the left panel. Thereby the shortest distance between two neighbors is given by 
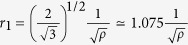
. The other distances between further neighbors appear in the following sequence: 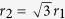, 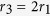, 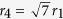, 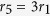, and 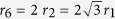.

The pair distributions are plotted in Fig. 3 for different values of 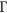. Data for colloidal systems are also provided in the moderate coupling Γ = 16.3 and 21) regime where equilibration is sufficiently fast to avoid the pinning of the sedimented particles on the glass substrate^8^, see Methods for more information. Two typical regimes are observed. The so-called hexatic phase is found at moderate dipolar coupling (here Γ = 16.3 and Γ = 21) and a solid phase emerges at high dipolar coupling (here Γ = 42.2 and Γ = 53.5). The agreement between experiments and MC simulations is remarkable and constitutes a decisive discovery bridging two well distinct scales and systems exhibiting the very same ordering. It is interesting to see that the excellent agreement between experiments on grains and simulations holds in situations where 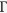 is roughly doubled, going from about 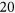 to about 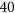, but also when it is varied by relatively smaller increments (roughly 20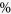). More specifically, in the regime of high dipolar coupling pronounced peaks in 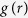 set in at distances of nearest neighbors of a triangular lattice, see Fig. 3. At intermediate dipolar coupling the ordering still persists, but the peaks now get broader and even merge. The latter feature is especially vivid for the second and third peaks located at 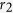 and 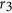, respectively, see Fig. 3.

To further investigate the ordering of the magnetized particles, we have monitored the bond-orientational function 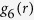 defined as follows


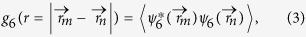


with


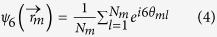


denoting the local sixfold bond orientational order parameter, where 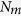 is the number of nearest neighbours of particle 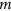 and 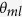 is the angle formed between the bond linking particles 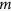 and 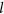 and an arbitrary prescribed reference axis.

**Figure 4:**
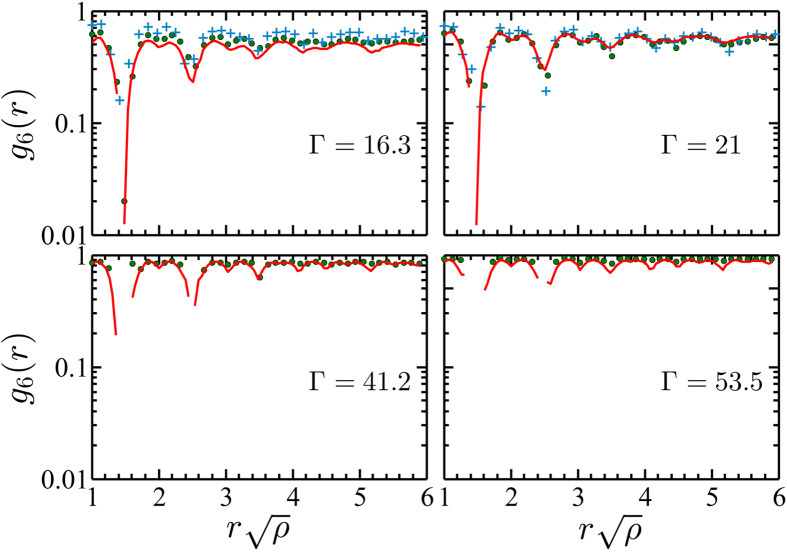
Bond-orientational correlation functions for different values of the magnetic coupling parameter 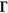
. Full lines correspond to MC simulation data, and dots (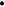) and stars (+) represent granular and colloidal experimental data, respectively.

The bond-orientational functions are plotted in Fig. 4 for different values of 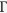. At moderate coupling, good agreement between the three approaches (granular, colloidal, and simulated systems) is achieved. Upon increasing 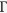, 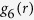 increases and becomes close to unity at 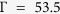. Clearly, at high dipolar coupling (here Γ = 42.2 and Γ = 53.5), 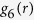 tends towards a constant at large separations whose value increases with 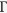, in accordance with the Kosterlitz-Thouless-Halperin-Nelson-Young (KTHNY) theory predicting (asymptotically) a constant value in the crystalline phase. In the hexatic phase (here Γ = 16.3 and Γ = 21), the KTHNY theory would predict an algebraic decay. However, given the limited finite number of beads in the granular systems, a quantitative comparison with the KTHNY theory, that holds for infinite systems, is restricted.

## Discussion

We have shown that Brownian motion can be induced in athermal systems such as granular media following a recently introduced technique^13^. In this study, the crucial energy/entropy interplay is contained in the single universal dimensionless parameter 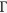. The ordering in the granular system matches quite quantitatively that found experimentally and numerically in colloidal systems. This constitutes good news for the Soft Matter community, since setting up experiments on (dry) granular systems is much easier and convenient (e.g. considerably faster equilibration^13^) than on (wet) colloidal suspensions^14^. This being said, we are aware that our approach is only relevant for two-dimensionnal granular systems since gravity would cause inhomogeneity in the third dimension in contrast to colloidal systems. Moreover, the effective temperature of our granular media is well defined because dissipative collisions between particles are virtually nonexistent due to repulsive interactions and/or low particle concentration of the system. This new experimental approach involving forced Brownian motion in macroscopic granular systems can be extended to study 2D binary systems^7^,^6^. Quite generally speaking, a wide range of repulsive colloidal systems can be mapped onto granular ones. For the sake of the present paper, magnetic dipolar interactions (in contrast to electrostatic forces for instance^15^) were the example of choice due to the ease of tailoring their strength. Nonetheless, particles such as Janus spheres^16^, presenting one attractive hemisphere, could also be mimicked by macroscopic beads whose self assembly process could be conveniently monitored. Another great advantage of fundamental importance is that reaching ground state situations (i.e. very high coupling value) is straightforward for granular media where induced noise can be merely suppressed by turning off the sources. Since both agitation and magnetic interaction could be tuned precisely and rapidly, the present granular system can be used to study the pathways of relaxation during ultrafast quenching^7^.

## Methods

### Experimental

#### Granular media

A set of 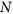 soft-ferromagnetic beads of diameter 1 mm are confined in a 2D hexagonal cell of diameter 3 cm. The hexagonal cell is horizontally excited by two perpendicular loudspeakers (see Fig. 1) generating sinusoidal vibrations with an amplitude of 100 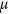
m, a frequency of 35 Hz, and without phase difference. Under these conditions, the beads respond as Brownian particles, i.e. erratic displacements are observed.The system is placed in a vertical and homogeneous external magnetic field 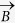. Considering that each bead has the same dipolar moment 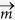, the beads interact via a repulsive pair potential


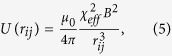


with 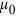 denoting the vacuum permeability and 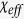 the effective susceptibility relating the dipolar moment of one bead with 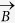 such that 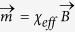. The interaction is well controlled by adjusting the external magnetic field strength. More specifically by defining 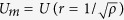, with 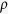 standing for the particle area density, the magnetic coupling can be fully characterized by the dimensionless 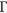 parameter given by Eq. (1).

In order to obtain reproducible initial conditions, beads are placed in a perfect hexagonal configuration. In order to form a finite triangular lattice, the number of beads 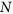 must satisfy the relation 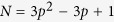 where *p* is the number of beads per outer edge. The experiment is performed here with N = 547 beads (*p* = 14). Before each measurement, the agitation and the external magnetic field are switched on for 200 seconds to allow the system to reach the equilibrium state. After this initialization process, a CCD camera records a series of images at a fixed rate of 10 frames per seconds during 100 seconds. A basic tracking method allows one to determine the position and the trajectory of each beads during the whole experiment. Observables are computed from these trajectories, see^13^ for details.

#### Colloidal suspensions

Superparamagnetic colloids of diameter 4.5  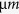 and mass density 1.7 × 10^3^ k*g*/m^3^ (Invitrogen) are dispersed in a Sodium Dodecyl Sulfate solution in order to prevent their aggregation. The suspension is filled in a cylindrical cell of diameter 5 mm glued on a glass slide. A 2D monolayer is obtained by the sedimentation of the particles on the glass/water interface. The large volume of water above the array of particles prevents any drift caused by thermal gradients or the disturbance of the air-water interface. Similarly to the experiments in granular media, the repulsive pair potential given by Eq. (5) is generated by an external magnetic field applied perpendicularly to the particle layer. The same definition of the typical repulsion at mean separation 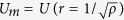 holds.

The magnetic interaction is increased in small steps followed by an equilibration time of several hours. After equilibration the particles coordinates are determined using an inverted microscope equipped with a CCD camera. Typically, the field of view 
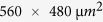
 contains about 1400 particles.

### Monte Carlo simulations

The very same geometry and number of particles in our experiments on granular media are employed in our numerical MC simulations. The constitutive (here point-like) particles interact via the pair potential


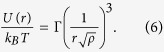


A standard MC procedure is executed to generate a canonical ensemble^17^. Typically 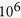 MC steps are used to equilibrate the system and 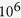 additional MC steps are conducted to gather statistics for the computation of the required observables. We have carefully checked that upon starting with a perfect lattice or a fully randomly generated configuration, identical properties are obtained within the statistical uncertainties. This guarranties that equilibrium is indeed reached. The experimental values of the effective susceptibilities 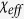 for granular and colloidal systems are deduced from the best matching in 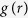 between simulations and experiments, similarly to the method employed by Zahn *et al.*^4^

## Additional Information

**How to cite this article**: Messina, R. *et al*. Quantitatively mimicking wet colloidal suspensions with dry granular media. *Sci. Rep.* **5**, 10348; doi: 10.1038/srep10348 (2015).
